# Confronting Pediatric Weight‐Based Cybervictimization: Time for Action

**DOI:** 10.1002/osp4.70073

**Published:** 2025-06-19

**Authors:** Silvia Zumaglini, Stephanie Fredrick, J. Kevin Thompson, Myles S. Faith

**Affiliations:** ^1^ Department of Counseling School, and Educational Psychology Graduate School of Education University at Buffalo Buffalo New York USA; ^2^ Department of Psychology University of South Florida Tampa Florida USA; ^3^ Center for Ingestive Behavior Research University at Buffalo Buffalo New York USA

**Keywords:** cybervictimization, obesity, schools, weight‐based victimization

## Abstract

Weight‐based victimization represents a critical challenge for youth, particularly those with obesity, and has been linked to a range of negative psychological, academic, and behavioral outcomes. While research has extensively examined in‐person victimization, the rise of digital platforms has given way to weight‐based cybervictimization, which remains understudied. This paper highlights the urgent need for research, prevention, and intervention strategies focused on weight‐based cybervictimization, emphasizing its harmful effects and its overlap with traditional forms of victimization. It identifies gaps in the existing literature, particularly regarding the inconsistent use of assessment tools and terminology in research on weight‐based victimization, and proposes the necessity for culturally relevant and validated measurement tools that accurately capture youth experiences. Finally, it aims to inform best practices for healthcare providers, educators, and parents by promoting strategies that effectively address and mitigate the impact of weight‐based victimization among youth.

## 1 Weight‐Based Victimization and Cybervictimization in Youth

Weight‐based victimization (WBV) refers to teasing and bullying targeting body weight [1] and is a significant problem for children, especially those with obesity. Research involving over 2000 adolescents identified WBV as the most common form of harassment, affecting 35.3% of participants, closely followed by race‐based harassment at 35.2% [2]. The negative impact of WBV is well‐documented, showing associations with depression, anxiety, low self‐esteem, suicidal ideation, poor academic performance, and lower motivation for physical activity [1, 3, 4]. In a sample of 394 students who experienced WBV, 40%–50% reported feelings of sadness, depression, body dissatisfaction, anger, and fear [5]. Furthermore, WBV is associated with lower physical activity and binge eating [5], which may further exacerbate obesity. Meta‐analyses have found that youth with overweight or obesity are more frequently bullied than healthy‐weight peers [6, 7], contributing to the “pain of obesity” [8].

An important caveat to this literature is its primary focus on in‐person, face‐to‐face, “school yard” experiences. This decades‐long research evolved before the explosion of contemporary social media, the internet, and related online platforms that dominate youth lives today. With this has come a spike in “cyberbullying,” defined as intentional harm or harassment directed at individuals through electronic platforms, including social media [9]. The experience of being targeted by these behaviors is referred to as cybervictimization, which in youth has been shown to prospectively predict internalizing problems, depression, anxiety, and suicidality [10, 11, 12, 13]. Moreover, cybervictimization impacts educational outcomes, including poorer school attendance and academic difficulties [9]. These findings underscore the interweaving detrimental effects of cybervictimization on children's psychological, behavioral, and academic development. Notably, research suggests a significant overlap between in‐person and online victimization, with youth who experience traditional victimization being likely to be targeted online as well [14]. This highlights the importance of addressing both forms of victimization in research and interventions.

Surprisingly, few studies have focused on cybervictimization specifically targeting weight, obesity, or obesity‐related behaviors in children, that is, “weight‐based cybervictimization” [15]. This is a significant knowledge gap in light of emerging data. In a recent study of 452 adolescents aged 11–17 years, 33% reported experiencing weight‐based victimization online [15], with prevalence rates of 45% and 60% among youth with overweight and obesity, respectively. Puhl et al. [15] reported that up to 60% of adolescents seeking weight loss have experienced weight‐based victimization online, and social network analyses indicate that attacks targeting individuals with overweight or obesity on social media are common and predominantly negative and derogatory [16, 17]. Adolescents were more likely to victim blame when the individual being cyberbullied was overweight rather than being thinner [18]. Perhaps not surprisingly, adolescents with obesity report avoiding certain social media platforms and refraining from presenting their bodies online as a form of self‐defense [19]. Victims of appearance‐related cybervictimization, including those targeted for weight and body shape, report more experiences of body shame than those targeted for other reasons [20].

These dynamics are not only supported by empirical data, but also reflected in public discourse. Many widely reported cases, including adolescents and young public figures who experienced cybervictimization related to their weight and body image, sometimes culminating in disordered eating or even suicidality [21, 22, 23], underscore the tangible and often devastating impact of this form of harassment. One tragic example that brought national attention to the dangers of weight‐based cyberbullying is the case of Brandy Vela, an 18‐year‐old who died by suicide after enduring months of online harassment about her weight [21]. Her story is one of several widely reported cases highlighting the life‐threatening risks associated with weight‐based victimization. This issue is clinically significant for many children and adolescents, highlighting the need for much more research and greater professional education.

## 2 Challenges in Terminology and Measurement

To move this area of study forward, efforts should focus on achieving greater clarity and consensus regarding *measurement*. This presents both a challenge and opportunity, because assessment needs to be done in a way that achieves psychometric rigor while still using language that is meaningful to children and adolescents (as participants) and also being feasible and flexible for health care providers in clinics and educational settings (for assessment and possible intervention). Indeed, the terms “weight‐based cyberbullying,” “body shaming,” “weight‐based teasing,” and “weight‐based harassment” have been used in studies, sometimes interchangeably. The term “body shaming,” for example, has been sometimes used without a clear definition [24]. While body shaming, like weight‐based cyberbullying, involves negative body‐related comments or insults, cyberbullying is often characterized by prolonged and repeated actions by the same perpetrator, suggesting that body shaming can evolve into cyberbullying [24].

Another challenge with terminology is that youth often avoid using the term “cyberbullying,” associating it with high severity. Instead, some research suggests they prefer terms such as “online conflict” or “drama,” even when describing situations and consequences that fit the definition of cyberbullying [19, 25]. Some adolescents feel more comfortable using terms like “drama” to describe bidirectional interactions and avoid labeling themselves as victims, a concept frequently used by adults [19]. The term “cyberbullying”, let alone “weight‐based cyberbullying”, may not always resonate with adolescents, who often describe cyberbullying based on the extent of harm caused rather than the raw number of exposures or occurrences. Clinicians and researchers might benefit from alternative terminology that is more closely aligned with concepts related to the experiences and every‐day language of children and adolescents [19].

Fortunately, new instruments have been validated and are yielding fruitful results. Puhl et al. [26] measured weight‐based victimization using a 19‐item instrument that showed solid reliability (*α* = 0.94), which included items on verbal teasing, physical aggression, and relational victimization, with some items focused specifically on online platform exposure. Another significant contribution is from Lessard and Puhl [15], who developed a specific measure for weight‐based cybervictimization, marking a crucial step forward in this field. This measure includes a definition of bullying, followed by eight items that assess experiences such as name‐calling, insults, exclusion, rumor‐spreading, threats, and other harmful behaviors by peers online or via text, specifically due to body weight. An important need in this field is measurement tools validated in culturally diverse populations, as well as studies including minority and marginalized youth. There may be intersectionality among obesity status, sex, and race regarding the impact of weight‐based cybervictimization [2]. It is noteworthy that the Perception of Teasing Scale [27], generally considered to be the “gold standard” questionnaire for assessing traditional weight‐based victimization, has been used successfully with diverse populations over many years [28] and thus may help inform new measures of weight‐based cybervictimization. Qualitative methods may also prove helpful for instrument development and capture cultural and individual differences [25].

## 3 Call to Action: Next Steps for Research and Practice

Understanding the impact of weight‐based cybervictimization is important for professionals working in healthcare and educational settings with youth with obesity. This may be challenging given the knowledge gap of many health care professionals, that is, the generational divide in technology literacy. Fortunately, there are some excellent resources available to support professionals (see Table 1 for sample resources). Healthcare providers are uniquely positioned to serve as key intervention agents, especially since youth with obesity may experience teasing or bullying not only from peers but also from teachers and family members [29]. Furthermore, given the known overlap between traditional and cyber victimization [14], youth who experience weight‐based victimization are likely to also face weight‐based cybervictimization, necessitating prevention and intervention strategies that target both forms simultaneously.

**TABLE 1:** Sample resources for addressing weight‐based cybervictimization in clinics, schools, and educational settings.

Resource	Information
Common Sense Education https://www.commonsense.org/education	Offers free digital well‐being and digital citizenship lessons for Grades K‐12, including lessons on cyberbullying, “digital drama,” and online hate speech
Cyberbullying Research Center https://cyberbullying.org/	Provides resources for educators, healthcare providers, parents, and students on cyberbullying prevention and intervention
Learning for Justice https://www.learningforjustice.org/	Provides free lesson plans on bias‐based bullying and cyberbullying, and training for educators on “speak Up at School” to create a safe and inclusive school climate
Obesity Action Coalition https://www.obesityaction.org/	Provides science‐based education on obesity and its treatment and advocates to eliminate weight bias
Obesity Medicine Association https://obesitymedicine.org/	Offers resources and evidence‐based articles on obesity. Includes clinical practice statements offering guidance, including how to support youth experiencing victimization and weight stigma
Stop Weight Bias https://stopweightbias.com/	Offers educational tools and information on weight bias and obesity
UConn Rudd Center for Food Policy and Health https://uconnruddcenter.org/	Offers resources on weight bias, weight stigma, and weight discrimination, along with strategies to address these issues

Healthcare and education professionals can be trusted and “safe” allies available to support youth experiencing weight‐based cybervictimization [29]. They can do this through supportive, compassionate, non‐stigmatizing communications, screening for experiences of victimization, and educating families about weight stigma [30, 31]. Teachers also can play an important role. Research shows that students who view their teachers as proactive in addressing weight‐based victimization experience fewer negative impacts on their academic performance [1]. Enhancing school‐based anti‐bullying programs to specifically address weight‐based victimization and target those at higher risk, such as students with overweight or obesity [4], could further help mitigate its negative impact on academic performance and overall well‐being. Notably, adolescents attending a national weight‐loss camp identified stronger policies targeting weight‐based victimization as a key strategy to help them cope [18, 32]. Additionally, explicitly including body weight in school anti‐bullying policies has been linked to lower weight‐related biases among educators, potentially encouraging greater intervention [33].

In addition to clinicians and educators, parents play a critical role in preventing weight‐based victimization. By fostering supportive environments and modeling non‐stigmatizing attitudes, parents may help reduce the negative psychological and behavioral consequences of weight‐based victimization. Himmelstein and Puhl [32] found that parental support was identified by most weight‐loss–seeking adolescents as the most important factor in coping with weight‐based victimization. More broadly, parental warmth, that is, behaviors that promote acceptance and emotional support, has been consistently associated with a lower likelihood of cybervictimization [34]. Additionally, while restrictive parental control strategies (such as limiting internet access) show weak associations with reduced victimization, active mediation strategies, such as open discussions about internet use and collaboratively setting rules, are more strongly linked to lower involvement in cybervictimization [34, 35, 36]. Parental communication about weight may also play a critical role: research indicates that parental weight talk may have negative implications for adolescents' self‐perceptions, whereas conversations focused on healthy behaviors without weight‐based criticism may be more supportive [36]. Empowering parents with resources and education to adopt these supportive strategies may enhance prevention efforts.

Addressing the harmful consequences of weight‐based cybervictimization requires focused research to guide effective prevention and intervention efforts. Future studies should prioritize longitudinal designs to examine the long‐term impact of weight‐based cybervictimization and assess the effectiveness of prevention strategies over time. Additionally, school‐based intervention studies targeting both traditional and cyber forms of weight‐based bullying can inform the development of targeted, evidence‐based programs within educational settings. Further research should also focus on validating assessment tools across culturally diverse populations and ensuring consistent, developmentally appropriate terminology that accurately captures youth experiences of weight‐based victimization. Controlled intervention studies ultimately will inform best practices for schools, clinics, or family settings to combat the negative effects of weight‐based cybervictimization among youth with obesity, improving physical health, emotional well‐being, and academic success. Confronting weight‐based cyberbullying also might help augment interventions to reduce childhood obesity—perhaps through the any of the aforementioned pathways if not others—which would be a “win” for public health and disease prevention.

## Conflicts of Interest

The authors declare no conflicts of interest.
